# Dual-lifetime referencing (DLR): a powerful method for on-line measurement of internal pH in carrier-bound immobilized biocatalysts

**DOI:** 10.1186/1472-6750-12-11

**Published:** 2012-03-28

**Authors:** Caterina Boniello, Torsten Mayr, Juan M Bolivar, Bernd Nidetzky

**Affiliations:** 1 Austrian Center for Industrial Biotechnology (ACIB), Petersgasse 14, A-8010 Graz, Austria; 2 Institute of Biotechnology and Biochemical Engineering, Graz University of Technology, Petersgasse 12, A-8010 Graz, Austria; 3 Institute of Analytical Chemistry and Food Chemistry, Graz University of Technology, Technikerstraße 4, A-8010 Graz, Austria

## Abstract

**Background:**

Industrial-scale biocatalytic synthesis of fine chemicals occurs preferentially as continuous processes employing immobilized enzymes on insoluble porous carriers. Diffusional effects in these systems often create substrate and product concentration gradients between bulk liquid and the carrier. Moreover, some widely-used biotransformation processes induce changes in proton concentration. Unlike the bulk pH, which is usually controlled at a suitable value, the intraparticle pH of immobilized enzymes may deviate significantly from its activity and stability optima. The magnitude of the resulting pH gradient depends on the ratio of characteristic times for enzymatic reaction and on mass transfer (the latter is strongly influenced by geometrical features of the porous carrier). Design and selection of optimally performing enzyme immobilizates would therefore benefit largely from experimental studies of the intraparticle pH environment. Here, a simple and non-invasive method based on dual-lifetime referencing (DLR) for pH determination in immobilized enzymes is introduced. The technique is applicable to other systems in which particles are kept in suspension by agitation.

**Results:**

The DLR method employs fluorescein as pH-sensitive luminophore and Ru(II) tris(4,7-diphenyl-1,10-phenantroline), abbreviated Ru(dpp), as the reference luminophore. Luminescence intensities of the two luminophores are converted into an overall phase shift suitable for pH determination in the range 5.0-8.0. Sepabeads EC-EP were labeled by physically incorporating lipophilic variants of the two luminophores into their polymeric matrix. These beads were employed as carriers for immobilization of cephalosporin C amidase (a model enzyme of industrial relevance). The luminophores did not interfere with the enzyme immobilization characteristics. Analytical intraparticle pH determination was optimized for sensitivity, reproducibility and signal stability under conditions of continuous measurement. During hydrolysis of cephalosporin C by the immobilizate in a stirred reactor with bulk pH maintained at 8.0, the intraparticle pH dropped initially by about 1 pH unit and gradually returned to the bulk pH, reflecting the depletion of substrate from solution. These results support measurement of intraparticle pH as a potential analytical processing tool for proton-forming/consuming biotransformations catalyzed by carrier-bound immobilized enzymes.

**Conclusions:**

Fluorescein and Ru(dpp) constitute a useful pair of luminophores in by DLR-based intraparticle pH monitoring. The pH range accessible by the chosen DLR system overlaps favorably with the pH ranges at which enzymes are optimally active and stable. DLR removes the restriction of working with static immobilized enzyme particles, enabling suspensions of particles to be characterized also. The pH gradient developed between particle and bulk liquid during reaction steady state is an important carrier selection parameter for enzyme immobilization and optimization of biocatalytic conversion processes. Determination of this parameter was rendered possible by the presented DLR method.

## Background

Establishing a biocatalytic process on a carrier-bound immobilized preparation of the used enzyme is desirable for several reasons. A heterogeneous biocatalyst is readily recovered from the liquid bulk, hence is multiply re-usable [1]. Reactions are readily implemented in continuous or repeated batch modes of operation. Enzymes are often stabilized significantly against inactivation by (multipoint) attachment onto a carrier surface, substantially enhancing their total turnover in the reaction process [2-4]. However, the carrier and the immobilization procedures require extra expenditure in biocatalyst preparation; in addition, carrier-bound enzymes are usually (much) less active than their native soluble counterparts. The observed loss of enzyme activity comprises a *real *and an *apparent *component [1,5]. In real inactivation, a combination of diverse denaturation processes occur, likely resulting from attachment of the enzyme to the carrier. These processes include conformational distortion, chemical modification and improper orientation of the enzyme on the solid surface [3,6]. Unfortunately, causal relationships between enzyme immobilization and denaturation at the molecular level are, to date, not understood enough. Apparent inactivation refers to diffusional limitations and their consequences on the observable enzymatic rate, and arises from two factors. First, if the characteristic times for mass transport into and out of the porous carrier are of the same order of magnitude as the characteristic time for the enzymatic reaction, the overall conversion rate for the immobilized preparation will be lower than that for the native enzyme used in homogeneous biocatalysis [4,5,7]. Second, diffusional limitations may induce concentration gradients between bulk liquid and the interior of the carrier, which may steer the microenvironment of the immobilized enzyme away from optimal for activity, stability or both [5]. Moreover, the chemical transformation may be itself negatively affected with respect to selectivity or equilibrium position under these conditions [4,7]. Knowledge-based selection of a carrier whose geometrical properties (particle size, pore size) support optimum performance of the enzymatic reaction by attenuating the diffusion effects would facilitate development of efficient heterogeneous catalysts for applied biocatalysis.

We consider the case in which the enzymatic transformation proceeds with production or consumption of protons. This situation applies to a diverse range of conversions catalyzed by esterases, lipases, and some amidases of widespread use in the biocatalysis [1,2,5,7]. In previously published studies, kinetic modeling has been used to delineate spatiotemporal features of pH gradient formations in immobilized enzymes [8-10]. Results of these studies suggest that diffusional limitations may result in a substantial difference in the proton concentration between carrier and bulk. However, only a few studies have experimentally determined the internal pH of immobilized enzymes. Kasche, Spiess and coworkers published significant measurements of the pH drop in carrier resulting from enzymatic conversion of penicillin G using the pH-responsive fluorophore fluorescein isothiocyanate (FITC) [7] and compared their data to predictions from a reaction-diffusion model. In their method, change in FITC luminescence intensity was related to the change in the proton concentration. However, intensity-based measurements may be strongly affected by drifts in the optoelectronic assemblage (light sources and light detectors) and by variations in sensor layer properties (dye concentration or thickness) [11]. Moreover, large noise in the recorded signals may complicate the analysis of stirred or agitated suspensions.

Combining the pH indicator with a reference dye and measuring emission or excitation at two different wavelengths may overcome some of these drawbacks. Referenced intensity measurements have been performed in two previous studies of heterogeneous biocatalysts. Internal proton concentration was determined by confocal laser scanning microscopy, and the enzyme was fixed on either porous beads [12] or in a membrane [13]. The ability to record pH gradients within the carrier material at spatial resolution is a clear advantage of this method. However, light scattering and reflection are not referenced because luminescence is measured at two wavelengths [14]. Furthermore, the required equipment is expensive and not suitable for the study of particles in suspension.

Lifetime measurements of indicators whose lifetime depends on pH are highly accurate because lifetime is independent of intensity or wavelength interferences [14]. Spiess et al. [15] evaluated the diffusion of propionic acid into hydrogels by measuring the pH-dependent lifetime of resorufin using confocal laser scanning microscopy and pulsar excitation. Kuwana et al. [16] applied a frequency domain proton migration technique to measure the phase shift and thus the lifetime of a pH-sensing fluorophore, carboxy seminaphthofluorescein-1 immobilized on poly(ethylene glycol) microparticles. However, lifetime measurements of fluorescent pH indicators in the nanoseconds range require sophisticated optoelectronic equipment [14].

Given the critical limitations of the above-described approaches in the design, selection and optimization of enzyme immobilizates, we here present an alternative method for pH determination in enzyme carriers. The method is based on a procedure known as dual lifetime referencing (DLR) [17,18], previously applied in the development of optochemical sensors for pH, oxygen, chloride, copper and ammonia [16-20]. DLR utilizes a pair of luminophores, one being the pH indicator and the other the reference standard. The method relies on simultaneous excitation of the indicator and the reference and on measuring the overall phase shift resulting from the ratio of the two intensities [18]. DLR application requires that several criteria are satisfied: (1) that reference and indicator have large different decay times; (2) that spectral properties of reference are not affected by the sample; (3) that the excitation spectra of indicator and reference overlap (enabling both luminophores to be excited at a single band of wavelength), and (4) that the emission of both luminophores can be detected at a common wavelength or band of wavelength using a single photodetector. The result is a self-referenced measurement that is unaffected by optical system interferences. Specific details of the principle of measurement are described in Huber et al. [17].

**Figure 1:**
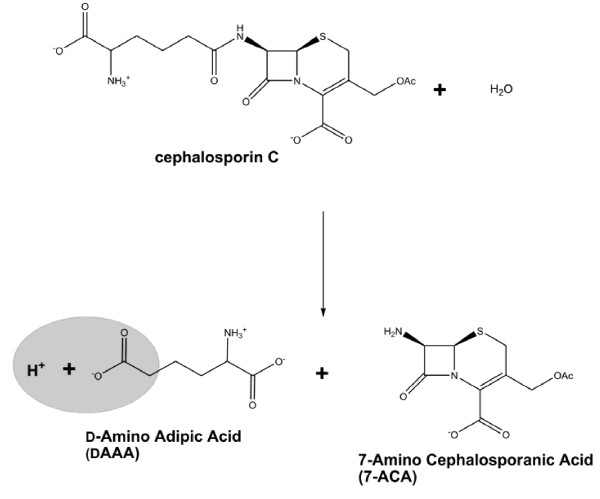
**Cephalosporin C hydrolysis catalyzed by CCA**. Formation of the DAAA product results in release of the molar equivalent of protons. In the case of immobilized CCA, proton formation may lead to a drop in intraparticle pH relative to the external pH in bulk.

The pH dependence of the phase shift is utilized for pH determination. Phase-shift data are relatively independent of particle movements and can thus be collected flexibly and at useful signal-to-noise ratio from a wide range of reaction systems including the common stirred tank. Reaction time course monitoring enables measurements to be completed in a time-resolved manner, as required. Moreover, the required instrumentation is inexpensive and easy to handle. The chosen procedural set-up provides information about the average pH inside the immobilizate, but does not elucidate the spatial resolution across characteristic dimensions of the carrier. However, biocatalytic process development with immobilized enzymes typically involves comparative evaluation of different process options. Because of the importance of the time required to complete this development, the applied analysis methods must balance adequate throughput with quality and information content of the acquired results. The DLR approach would seem to present a practically useful compromise, as shown in our recent report [21]. In the present paper, we describe details of the method and its application to immobilized enzyme characterization. The hydrolysis of cephalosporin C to 7-amino cephalosporanic acid (7-ACA) and D-α-amino adipic acid (DAAA) catalyzed by cephalosporin C amidase covalently tethered on epoxy-activated Sepabeads EC-EP is examined as a model system of industrial relevance (Figure 1).

## Results and discussion

### Determination of internal pH in enzyme immobilizates: Choice of materials, set-up of the analytical system, and sensor calibration

A DLR system was established using two luminophores of which one, fluorescein, displays pH-sensitive fluorescence behavior and the other, Ru(II) tris(4,7-diphenyl-1,10-phenantroline), abbreviated Ru(dpp), does not respond to pH and therefore serves as the reference. The lifetime of fluorescein is of the order of nanoseconds whereas that of Ru(dpp) is three orders of magnitude longer, in the microseconds range. Excitation and emission spectra of fluorescein overlap with the corresponding spectra of Ru(dpp) [11,14]) such that each luminophore can be excited at a common wavelength (470 nm) and can emit light within the same wavelength range (upwards of 550 nm). After excitation in the frequency domain, the fluorescence intensities of fluorescein and Ru(dpp) are converted into an overall phase shift, which is related to the proton concentration, as will be shown later.

**Figure 2:**
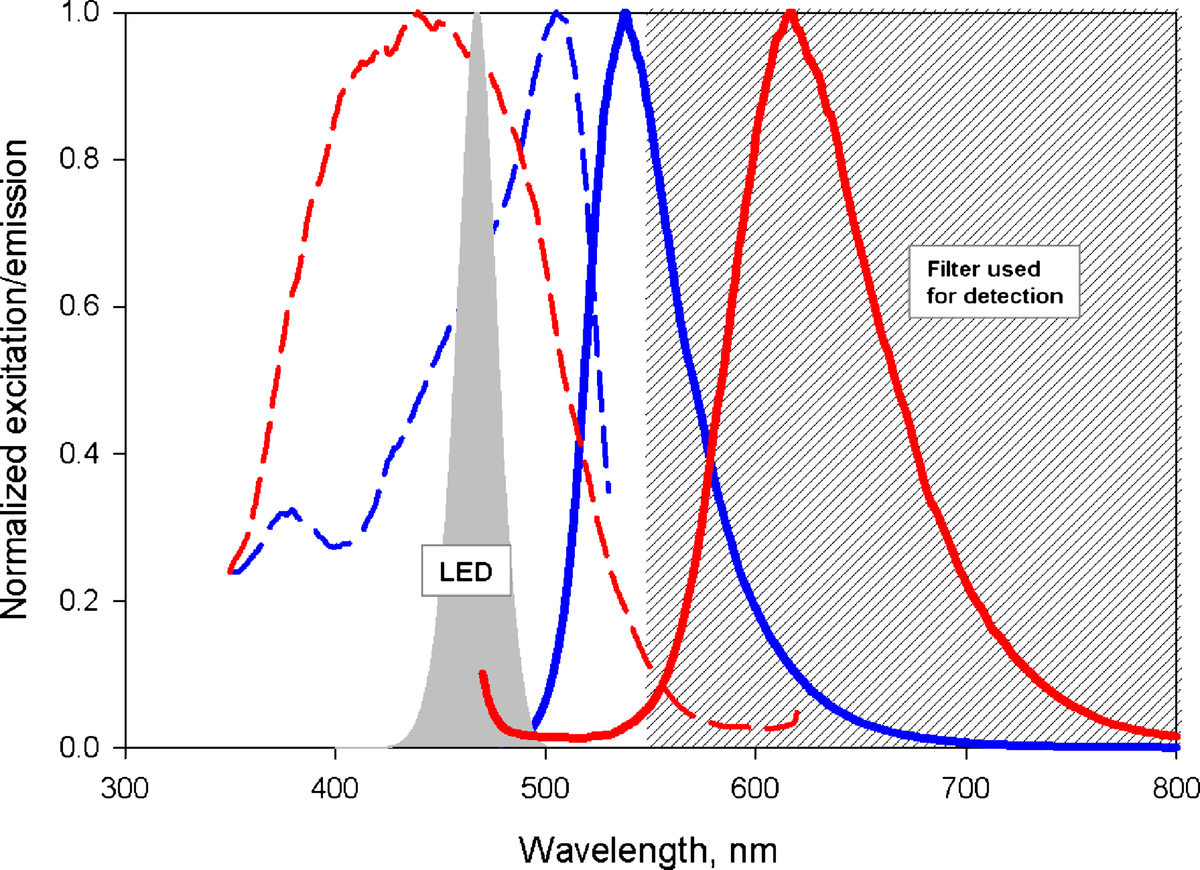
**Spectral properties of the luminescent dyes attached to Sepabeads and optical components used for DLR**. Excitation (dotted line) and emission (continuous line) spectra of Fluorescein (blue) and Ru(dpp) (red), and a schematic representation of the excitation source (LED) and the filter used for detection (grey area)

To apply DLR to pH measurement inside enzyme immobilizates, a means of attaching the two luminophores to the carrier used for immobilization must be established. A restriction considered was that functional surface groups by which the enzyme was attached to the carrier be not involved in luminophore binding. Sepabeads EC-EP were chosen for this study because they represent a class of polymer beads that have been widely used in applied biocatalysis as carriers for enzyme immobilization [6,22]. Sepabeads EC-EP are made of polymethacrylate and possess reactive epoxide groups on their surface. Considering the relative hydrophobic character of the Sepabeads EC-EP base material, we opted for highly lipophilic variants of fluorescein and Ru(dpp), namely 5(6)-N-octadecyl-carboxamidofluorescein and Ru(II) tris(4,7-diphenyl-1,10-phenanthroline di(trimethylsilylpropan sulfonate)). We aimed to physically incorporate the two luminophores into the polymeric matrix of Sepabeads EC-EP. The extra hydrophobic character of the luminophores was expected to prevent their partitioning from the carrier to the aqueous phase. Experiments in which labeled carriers were extensively washed with buffer confirmed this notion, showing that wash-out of either luminophore was not detectable. Excitation and emission spectra of the lipophilic luminophores were not affected by binding to Sepabeads EC-EP (Figure 2).

Since loading of luminophores into the carrier comprises physical incorporation into a hydrophobic matrix, the procedure should be compatible with various kinds of surface chemistries that have been exploited for covalent or non-covalent protein binding to Sepabeads and related carriers. Fluorescein and Ru(dpp) henceforth refer to the lipophilic variants of the luminophores.

The electronic assembly for DLR measurements consists of a dual-phase lock-in amplifier, a sine-wave modulated LED, an optical fiber, a detector and optical filters as previously described [17].

**Figure 3:**
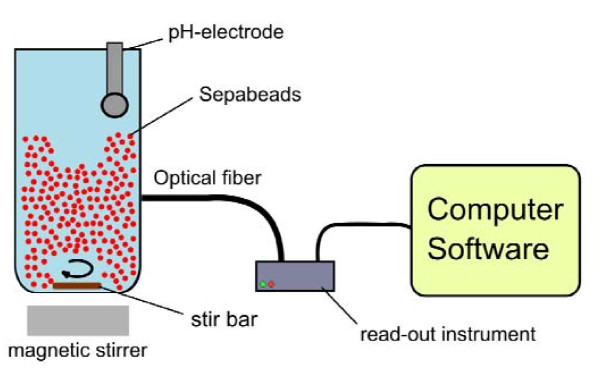
**General assembly for DLR measurements of internal pH in immobilized CCA**. The stirred reactor containing labeled CCA immobilizate was interfaced with an optical fibre cable connected to a commercial pH-Mini device (PreSens GmbH)

In this study we used a commercial compact device designed for the read-out of optical pH-sensors equipped with a 2-mm polymer optical fiber as shown in Figure 3. The spectrum of the excitation light is displayed in Figure 2. The spectra of the luminophores and the excitation light show a significant overlap. The emission is collected by the optical fiber and detected inside the pH-mini device. We emphasize the sensitivity of Ru(dpp) to O_2_, and that all of the reported measurements were performed in saturating air conditions at atmospheric pressure. Moreover, the chosen DLR system is sensitive to temperature and ionic strength. It is therefore imperative that calibration and later measurements be conducted under identical conditions.

**Figure 4:**
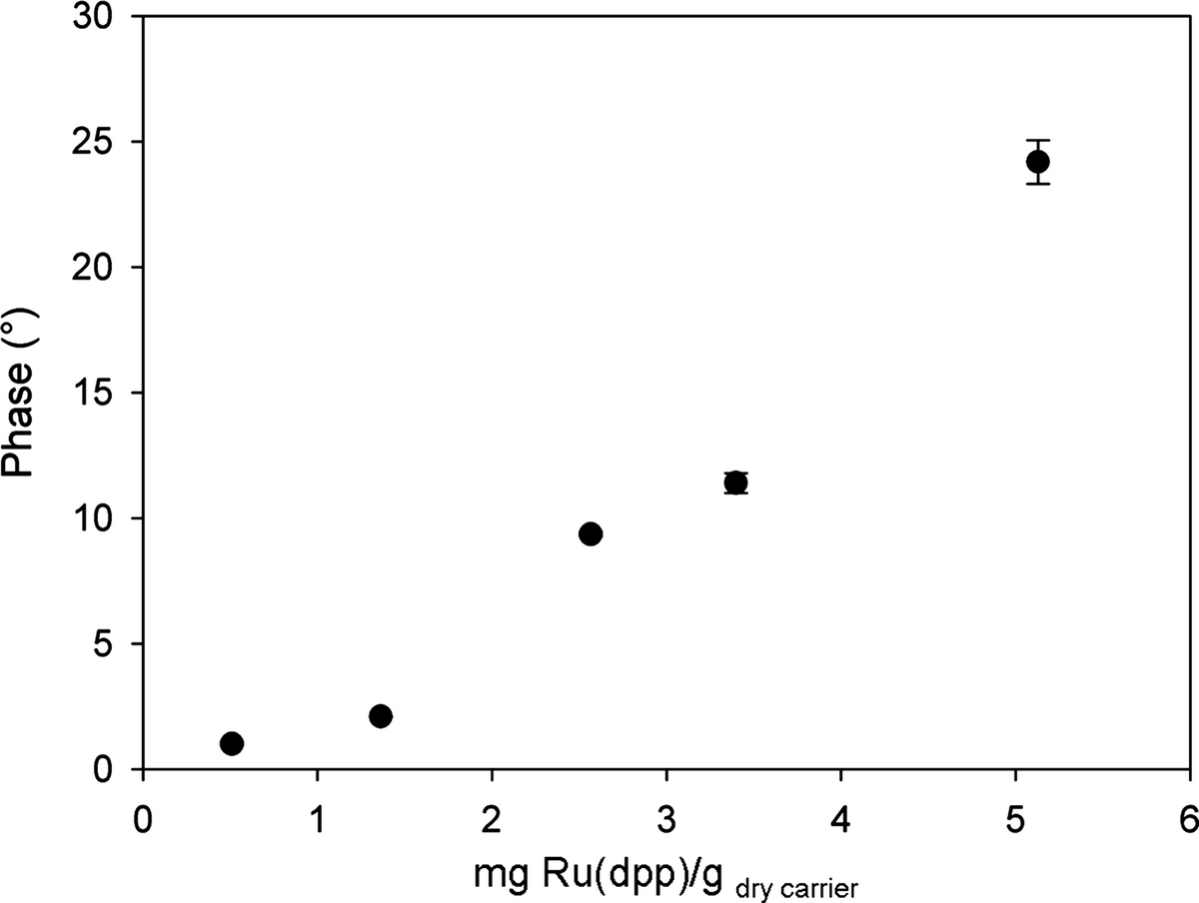
**Effect of Ru(dpp) loading (relative to fluorescein) on phase shift in DLR**. Experiments were performed in 50 mM PPB (pH 8.0) at 37°C. A constant fluorescein load of 2.5 mg/g _dry carrier _was applied. Modulation frequency was 45 kHz.

**Figure 5:**
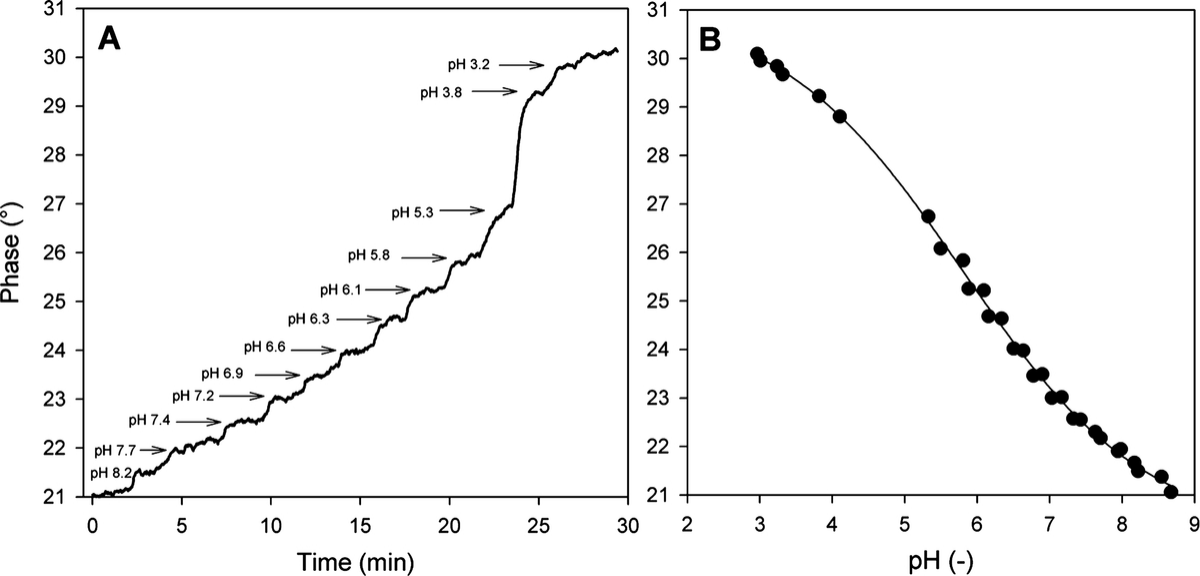
**Calibration of the intraparticle pH determination by DLR**. Panel A. Phase shift in response to pH. Panel B. Relationship between pH and phase shift. Experiments were performed in 50 mM PPB at 37°C. In panel B, symbols show data and the line is the fit to Equation 2, using the parameters in Table 1

It is clear from Equation (1), given by

(1) $$\text{cot}\Phi m=cot\Phi ref+\frac{1}{\text{sin}\Phi ref}\cdot \frac{Aind}{Aref}$$

where *A *is the amplitude of either indicator (*ind*) or luminophore (*ref*), and Φ is the phase angle of either the overall signal (m) or the luminophore (*ref*), that the overall phase shift depends on the relative fluorescence intensity of the two luminophores [18]. Therefore, under conditions in which the amount of fluorescein bound per unit mass of carrier is constant, a decrease in the amount of bound Ru(dpp) will reduce the phase shift. The effect of Ru(dpp) loading on the observed phase shift is displayed in Figure 4. Increase in mass ratio between Ru(dpp) and fluorescein introduced per unit mass of carrier was associated with substantial enhancement of the phase shift. At 37°C, loading of 2.5 mg fluorescein and 5 mg Ru(dpp) to each gram of dry carrier induced a phase shift of 21° at pH 8.5 and 30° at pH 3.0 (Figure 5A). At the applied frequency, this loading offers the most sensitive phase response to pH change.

Figure 5 shows how the phase shift dependence on pH was calibrated. Aliquots (50 - 100 μL) of 1.2 M HCl were added to 8 mL of a suspension of labeled Sepabeads (0.3 g) in 50 mM potassium phosphate buffer. The starting pH of the buffer was at 8.6. After mixing, the new pH in bulk was measured with a pH electrode, and the phase shift resulting from particle fluorescence was recorded for 1 min. An averaged value of the phase shift was used in the analysis (Figure 5A). Averaged results from two independent titrations are plotted in Figure 5B. The sigmoidal dependence of the phase shift on pH was fitted with the four-parameter model described by Equation 2 [19],

(2) $$\Phi =\frac{A_{\text{max}}-A_{\text{min}}}{1+10^{\left(pH-pK_{a}^{\prime }\right)/x}}+A_{\text{min}}$$

where Φ is the overall phase shift; *A*_max _and *A*_min _are limiting values of phase shift at high and low pH, respectively; *x *is a numerical coefficient related to the slope of the curve; and p*K*_a_' is the apparent p*K*_a _for the indicator/reference luminophore system immobilized on the enzyme carrier. Parameters of the model are summarized in Table 1. From the calibration curve in Figure 5B, pH is ideally determined in the range of about 4.0-8.0. It is of course preferable to operate as near as possible to p*K*_a_'. The standard error of the pH determination is typically of order 0.1 pH units.

**Table 1:** Parameters of the pH-phase calibration curve.

**Buffer**^**a**^	A_max _(°)	A_min _(°)	p*K*_a_'	x	**R**^**2**^
50 mM	31.1	20.2	5.80	1.26	0.998
	± 0.7	± 0.23	± 0.05	± 0.08	

Equation 2 was fitted to experimental data recorded at 37°C

^a^Ionic strength: 0.139 M

### Application of the DLR method to monitor spontaneous hydrolysis of cephalosporin C

**Figure 6:**
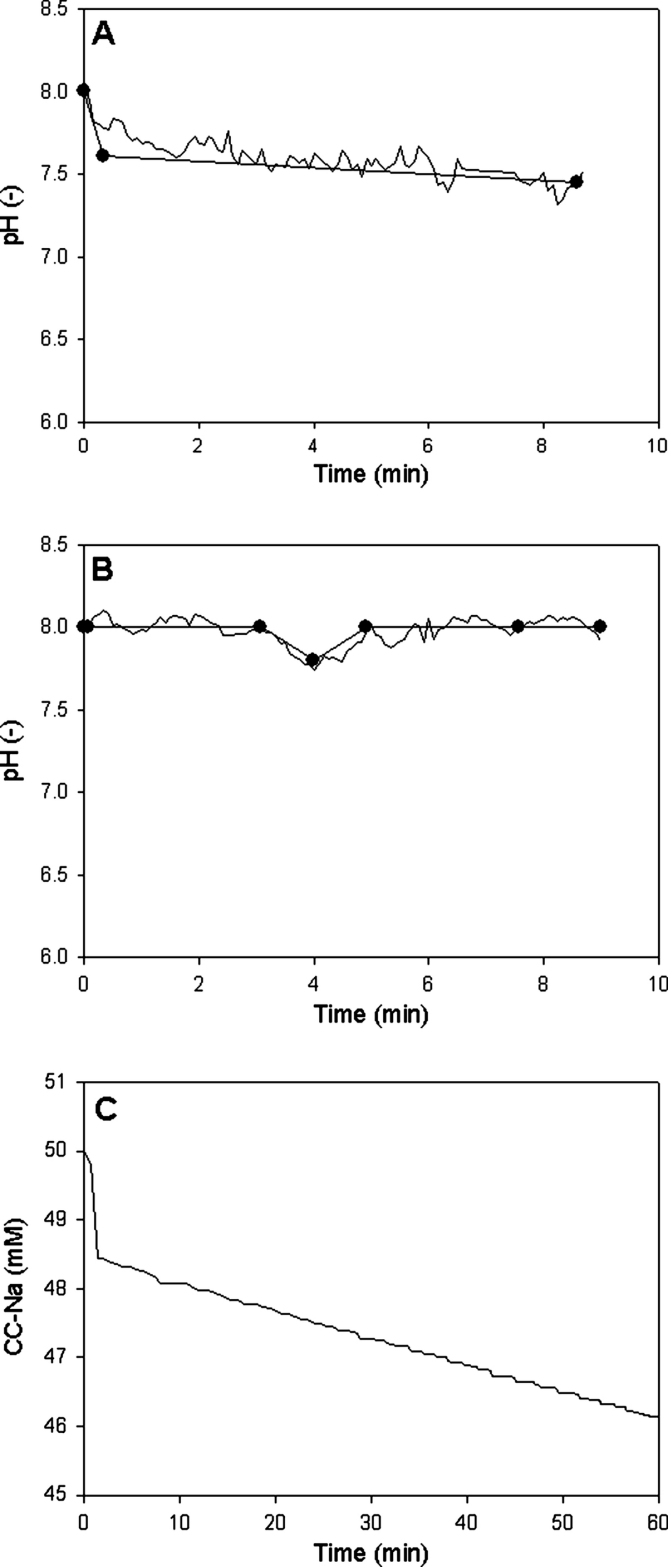
**pH changes in bulk and particle following addition of cephalosporin C, with no pH control (A) or with automated pH control in bulk (B), and spontaneous hydrolysis of cephalosporin C over time (C).** Experiments were performed in 50 mM PPB (pH 8.0) at 37°C. The symbols show pH measurements in bulk while the continuous line shows the particle pH. Labeled Sepabeads not containing enzyme were used in these experiments.

As a first example of the application of DLR in time-resolved measurement of intraparticle pH, we determined the acidification of Sepabeads EC-EP following incubation of the beads in the presence of cephalosporin C and in the absence of enzymatic conversion (conditions which induce spontaneous amide bond hydrolysis). Time courses for the pH change in carrier (measured by DLR) and bulk liquid (measured with a conventional pH electrode) are shown in Figure 6A. Both time courses were characterized by a rapid initial drop in pH followed by a slower, gradual decrease in pH with time. The time course of internal pH change in the slow phase agreed favorably with the corresponding pH change in bulk, indicating that the diffusion of protons into the particles was not significantly hindered; therefore, no pH gradient between bulk and particle emerged in the absence of a heterogeneous enzymatic reaction.

The experimental assembly for internal pH measurement necessitated that carriers were first suspended in buffer to initialize the phase shift under the given conditions (pH 8.0, 37°C). Cephalosporin C was then added at a high concentration (550 mM) to retain the overall volume change below 10%. To further minimize effect of spontaneous hydrolysis, the substrate solution was always prepared fresh, retained for a minimum time (≤ 30 min) at 4°C and adjusted to pH 8.0 prior to use. However, despite these precautions, the initial pH decrease was around 0.15 units following addition of substrate, too large to be neglected in the enzymatic conversion. Figure 6B is the result of an experiment performed with automated pH control in bulk. We observed a slight decrease in bulk and particle pH immediately following the addition of cephalosporin C to the suspension of labeled Sepabeads, which may be explained by prior compound hydrolysis in the stock solution of the substrate. After a short time, both pH values returned to the set pH.

The characteristic time for hydrolysis is the reciprocal pseudo-first order rate constant for decomposition of cephalosporin C in solution. Its value was determined as 1206 min from the slow reaction phase in a hydrolysis time course (Figure 6C). We can also calculate from Figure 6C that the amount of substrate hydrolyzed in the slow reaction phase was about 2.3 mM.

The following adjustments were therefore made for monitoring pH changes during the enzymatic reaction. The initial pH was read immediately following particle suspension of thoroughly mixed substrate. This pH was used to initialize the phase shift. The amount of substrate converted by slow auto-hydrolysis of substrate was subtracted from the overall conversion in the enzymatic reaction. Control experiments using buffer instead of substrate solution confirmed that the added liquid did not influence the recorded phase shift.

### Compatibility in the coimmobilization of luminophores and cephalosporin C amidase onto Sepabeads

Although care was taken that labeling of Sepabeads EC-EP with fluorescein and Ru(dpp) did not affect the epoxide groups that are primarily responsible for covalent attachment of enzyme to the carrier surface, it was important to confirm that carrier labeling did not grossly alter key parameters of the immobilization process. We therefore compared the protein binding and specific bound-protein amidase activity between Sepabeads treated with fluorescein or fluorescein/Ru(dpp) and untreated Sepabeads. The results, summarized in Table 2, indicate that the protein binding capacity was the same for all three carriers (within experimental error). The specific activity of immobilized amidase was slightly lower in labeled beads than in the untreated reference, although the observed differences were not significant. Therefore, if incorporation of the luminophores into the Sepabeads matrix influenced the activity of the bound enzyme, its effect was very small and not relevant to the experiments described below. However, the prevailing view is that luminophores may not in general be considered inert towards proteins and enzymes ([23]; JMB and BN, unpublished results 2011). Therefore, the extent of activity disruption in the presence of luminophore should always be investigated on a case-by-case basis.

**Table 2:** Effect of fluorescence labeling of Sepabeads on CCA immobilization

Labeling	Offered protein (mg/g dry carrier)	Bound protein (mg/g dry carrier)	Specific activity of bound CCA^a ^(U/mg)
Fluorescein	85	68	1.66 ± 0.06
Fluorescein + Ru(dpp)	85	68	1.50 ± 0.09
None	82	64	1.74 ± 0.17

^a ^Specific activity was measured using the spectrophotometric assay described in Boniello et al. [21]. Experimental conditions were: 40 mM cephalosporin C; 100 mM PPB (pH 8.0); 37°C

### Internal pH in Sepabeads containing immobilized amidase during conversion of cephalosporin C

**Figure 7:**
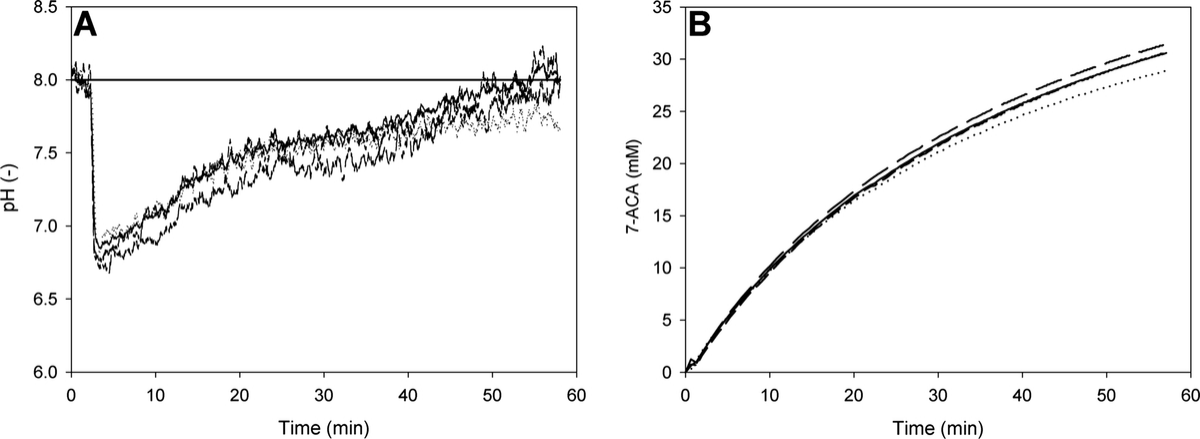
**Time course of internal pH during conversion of cephalosporin C by immobilized CCA**. Panel A. Internal pH measurements from four independent experiments. The bulk pH was constant at 8.0. Panel B. Formation of 7-ACA, determined indirectly from the amount of NaOH consumed for pH control. Experimental conditions were 0.65 mg/mL immobilized CCA; 50 mM cephalosporin C; 50 mM PPB (pH 8.0)

A batchwise conversion of 50 mM cephalosporin C (sodium salt; CC-Na) was performed in a total liquid volume of 8.8 mL (50 mM potassium phosphate buffer, pH 8.0) containing 0.3 g of immobilized enzyme preparation. The immobilizate was prepared as described in the Methods section using labeled Sepabeads EC-EP. The concentration of protein loaded into the reaction mixture was 0.65 mg/mL. The initial phase shift and the start bulk pH (following substrate addition) were determined as described above. Internal pH change was monitored from the change in phase shift as a function of the incubation time, for up to 60 min, while the bulk pH was automatically maintained at 8.0. As shown in Figure 7A, the internal pH decreased rapidly after substrate addition to its minimum of about 6.8. It then climbed gradually (Figure 7A) as the reaction progressed (Figure 7B), leveling out at around the bulk pH of 8.0 at the time of substantial slowing of the conversion rate. We emphasize that the rapid initial drop of the intraparticle pH by about 1.2 pH units (ΔpH = 8.0 - 6.8) cannot be solely attributed to the addition of partially hydrolyzed substrate (maximum ΔpH ≈ 0.3) (Figure 5A). The observed acidification of the Sepabeads must therefore arise from hydrolytic reaction catalyzed by enzyme immobilized in the carrier pores in the presence of strong diffusional limitations. The gradual approach of the intraparticle pH to that of the bulk solution (Figure 7A) can be attributed to a transition from initially mass transfer-limited reaction conditions to new conditions at the end of the conversion (in which enzymatic transformation was mainly rate determining). From the amount of NaOH base added by automatic titration, it was calculated that about 60% of the initial concentration of cephalosporin C had been converted into DAAA after 50 min of reaction, with concomitant release of protons (see Figure 1). Base consumption as an assay of cephalosporin C conversion was validated by comparing the proton concentration (measured indirectly via the added NaOH) and 7-ACA (measured with a spectrophotometric assay) produced in the initial phase of the reaction. With regard to this conversion, substrate depletion is clearly not solely responsible for the slowing down of the hydrolysis rate shown in Figure 7B, and it appears that other factors, perhaps product inhibition, also contributed to the observed rate retardation [24,25]. Indeed, this particular form of the hydrolysis rate curve is commonly observed in biotransformations catalyzed by both immobilized and soluble CCA, and it is usually attributed to inhibition or stability issues [24,25].

In the case of immobilized CCA, as presented here, mass transfer limitations and changes in internal conditions clearly play a crucial role in the reaction performance and final conversion. Considering that cephalosporin C amidase is much less active at around pH 7.0 than at pH 8.0 [21], quantitative measurements of the pH drop in the carrier are important information sources for biocatalytic process optimization. Based on the protein concentration in the reaction mixture, the specific reaction rate was determined as 1.48 ± 0.02 μmol/min/mg (proton release) or 1.45 ± 0.01 μmol/mg (7-ACA released), indicating an operational specific rate 3-fold less than the theoretical value. The observed decrease in intraparticle pH during conversion of cephalosporin C by the Sepabeads EC-EP immobilizate of the amidase (Figure 7A) suggests that removal of diffusional limitations through optimized carrier geometry should enhance reaction performance, by not only increasing the rate during the initial phase of the conversion, but also by affecting the operational stability of the enzyme. Mass transfer effects could be attenuated by widening the carrier pores. Increasing the bulk pH could constitute an alternative strategy of correcting the microenvironment of the immobilized enzyme, although the role of substrate auto-hydrolysis at high pH must also be taken into account. Moreover, time-resolved determination of intraparticle pH could provide a novel analytical technology tool with which to monitor the reaction and its progress *in situ*.

### Reproducibility of phase shift measurements and applicability

Figure 7 illustrates the results of three independent time-course experiments. The overall reproducibility of the experiment was high, with a calculated relative standard deviation of 4%. The standard deviation (S.D.) in the measured internal pH was ~0.1 pH units in the initial phase of the reaction. At the end of the conversion (after 60 min), the S.D. increased to ~0.2 pH units, which is still acceptable. Decline in the performance of the analytical system is readily explained by gradual displacement of the internal pH from that around p*K*_a_' (Table 1), which is optimal for DLR measurement. A pH of 8.0 already lies at the boundary of the region over which the dependence of phase shift on pH is linear (Figure 4B). Under these "non-linear conditions", an observed phase shift perturbation results in a proportionally larger change in pH, and data are therefore afflicted with higher standard error.

**Figure 8:**
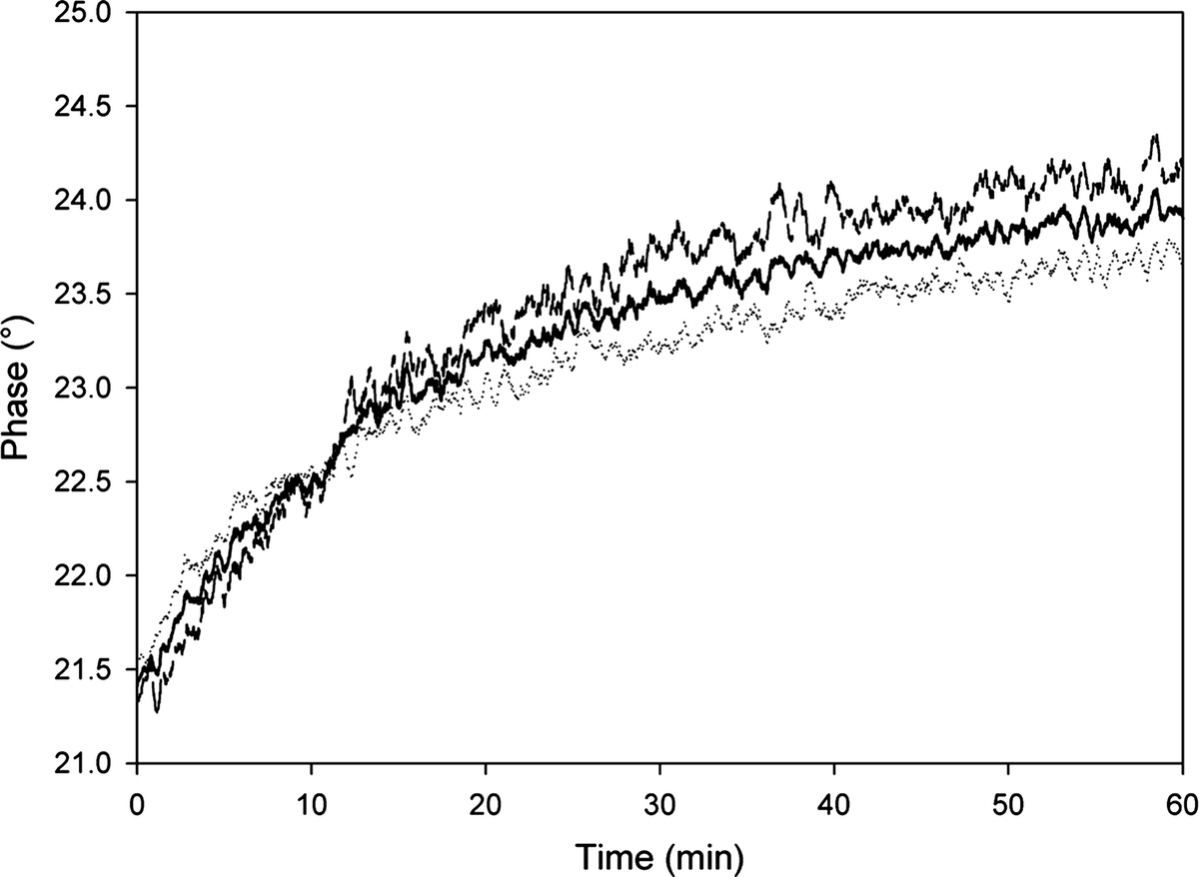
**Phase drift during DLR induced by photobleaching in three independent experiments**. Measurement conditions were: sampling rate, each second; LED intensity, 10%. Incubation conditions were as described for Figure 7, but without cephalosporin C.

During control measurements performed in conjunction with continuous determination of the internal pH in Sepabeads EC-EP (Figure 7), a substantial drift of the phase shift signal was observed. This drift increased as a function of the incubation time (equivalent to time of measurement). A 2.5° change in phase shift occurred within 60 min, corresponding to a large apparent displacement of pH by about 1.5 pH units (Figure 8). Leaching of luminophore molecules from the carrier or photobleaching of the indicator luminophore could be responsible for the observed signal drift, since either process would result in loss of luminophore intensity contributing to the phase shift, but neither would be referenced in DLR [14]. Release of lipophilic luminophores from carrier to aqueous buffer was carefully analyzed in controls. Given the absence of detectable leaching of bound luminophore in exhaustive washing experiments, insufficient stability of the carrier-bound DLR system appears unlikely. The alternative possibility, that photobleaching could cause signal drift, may be attributable to chemical decomposition of the pH indicator fluorescein triggered by the singlet oxygen generated by reaction of Ru(dpp) with molecular oxygen [19]. This idea was probed by changing the sampling frequency of the DLR measurement. The phase drift of 2.5°/h associated with a sampling period of one second decreased to 1.8°/h when sampling was performed every five seconds. However, the advantage of lowered signal drift was completely offset by the reduction in signal resolution that occurs with reduced sampling frequency. The resulting signals were too poor to allow dynamic averaging of the collected values. Reduction of photobleaching by minimizing light intensity (e.g. using just 10% of the available LED intensity) proved non-viable also. To overcome the problem of signal drift, control experiments lacking substrate were performed and the measured phase shifts were corrected using the relevant blank recordings (cf. Figures 5, 6 and 7).

A general comment on intraparticle pH measurement by DLR is that enhancement of signal intensities is expected to increase signal intensity, which in turn should stabilize measured values, improve resolution and thus lead to smoother responses. Higher intensities could be achieved by increasing the concentration of the labeled particles. The conditions of this study represent a compromise between reaction-system enzyme loading and labeled particle density.

## Conclusions

DLR is an analytical method that has been previously applied to various opto-chemical sensors. We have developed the method as a flexible and user-friendly means of determining the internal pH of carrier-bound immobilized enzymes. Such a means has previously been lacking. A major advantage of the method is its potential applicability to essentially all known types of enzyme reactors operating at multiple scales, irrespective of whether enzyme carriers are packed in beds or retained in suspension. The "hydrophobic incorporation" approach used for labeling the carriers with luminophores should be applicable to a variety of non-polar carrier materials independently of their functional activation. Signal drift caused by indicator luminophore photobleaching must be compensated by suitable calibration. The use of recently developed red light-excitable pH sensing materials [26], which exhibit chemical and photochemical robustness and are not affected by oxygen, could overcome this disadvantage.

## Methods

### Materials used

Sepabeads EC-EP (standard grade; specified particle size 150-300 μm; pore size 30-40 nm) were kindly provided by Resindion (Milan, Italy). Sepabeads are epoxy-activated polymethacrylate carriers widely used in enzyme immobilization studies. CCA was engineered from *Pseudomonas *sp. (strain SE83). More detailed information on the enzyme can be found in our previous paper [21]. The enzyme was obtained from Sandoz GmbH (Kundl, Austria) as technical-grade protein preparation; the batch used contained CCA with a purity of 60% and possessed a specific activity of 4.5 U/mg. The CCA was used without further purification. Fluorescein (sodium salt) was purchased from Sigma-Aldrich. Ruthenium(II) tris(4,7-diphenyl-1,10-phenanthroline) di(trimethylsilylpropane sulfonate) (abbreviated Ru(dpp)) was prepared according to Borisov and Klimant [27]. Cephalosporin C (sodium salt) was obtained from Sandoz GmbH at a purity of about 87%. All other materials were of reagent grade and obtained from standard suppliers.

### Spectral characterization of the labeled Sepabeads

Fluorescence excitation and emission spectra were acquired from a Hitachi F-7000 fluorescence spectrometer. Suspensions of Sepabeads labeled with Fluorescein or Ru(dpp) in 50 mM potassium phosphate buffer at pH 8 were measured in a cuvette equipped with a magnetic stirrer.

### Dual luminescence labeling

A stock solution of fluorescein (2 mg/mL in ethanol) was prepared and successively diluted with 100 mM potassium phosphate buffer (PBB), pH 7.2, to a final concentration of 5% (v/v). Approximately 1.6 g dry Sepabeads were incubated with 40 mL of this solution at room temperature, typically overnight (12-15 h), with gentle mixing in an end-over-end rotator operating at 15 rpm. The beads were then washed with 100 mM PPB (pH 7.2), deionized water, and 1.5 M PPB (pH 7.5). CCA was immobilized onto fluorescein-labeled beads as described later. Following immobilization, the beads were incubated in 40 mL Ru(dpp) solution, prepared by diluting stock solution (2 mg Ru(dpp)/mL ethanol) tenfold in 100 mM PPB, pH 7.2. Incubation period and conditions were 10 min at room temperature with mixing in the end-over-end rotator (15 rpm). Finally, the beads were washed thoroughly with 50 mM PPB, pH 8.0, and recovered by filtration through a 0.20 μm membrane filter.

### Measurement of internal pH

Phase shift measurements were performed using a lock-in amplifier (pH-mini, PreSens GmbH, Germany). This apparatus comprises a blue LED light source, a 2-mm polymer optical fiber as transducer with a polished distal tip and a photodetector (which collects emission after filtering at 550 nm). Measurements were carried out in the frequency domain, adjusting the modulation frequency to Ru(dpp) decay time (modulation frequency of 45 kHz). Time-resolved recorded phase shift was converted to pH using the previously described calibration.

### Experimental set-up

A 20-mL glass batch reactor was equipped with a jacket for temperature control from an external water bath. The temperature of the reactor volume was retained constant at 37°C. The reaction mixture was agitated with a stirrer. A titrator (T-50, Mettler Toledo, Greifensee, Switzerland) equipped with a pH electrode (Minitrode, Hamilton, Bonaduz, Switzerland) was used for stirring and bulk pH control (pH 8.0; 0.1 M NaOH). The stirring rate was selected to homogeneously distribute the enzyme immobilizate in the reactor (10% instrument setting). Approximately 0.3 g of wet labeled Sepabeads containing 19 mg of immobilized CCA per gram of wet carrier were suspended in 8 mL of 50 mM PPB (pH 8.0). The hydrolytic reaction was initiated by adding 800 μL of a 550 mM cephalosporin C solution previously kept at 4°C, and adjusted to pH 8.0. During the enzymatic reaction, the bulk pH and the amount of base added for pH control were recorded automatically. For phase shift detection of the labeled particles, the 2-mm polymer optical fiber connected to pH-mini was fixed inside the reactor (Figure 3). The amount of NaOH solution used represents the amount of cephalosporin C converted. At certain times, samples were collected for complementary measurement of 7-amino-cephalosporanic acid formation from cephalosporin C, using a colorimetric assay described in the literature [21].

### Enzyme immobilization

The method of Mateo et al. [28] was adopted with some simplifications. About 40 mL of enzyme solution (3.3 mg/mL) in 0.75 M PPB (pH 7.5) was added to 1.6 g dry labeled Sepabeads. The suspension was incubated at 18°C with gentle mixing (5 rpm) in an end-over-end rotator. After 24 hours, the immobilizate was washed twice with 20 mM PPB (pH 7.5), recovered on a 0.20 μm membrane filter and stored at 4°C for later use.

### Protein and activity assay

Protein concentration was measured using the Bio-Rad assay referenced against known concentrations of BSA in the range 0.10-1.0 mg/mL. Activity of free and immobilized CCA was measured via a reported spectrophotometric assay [29] with previously-described modifications [21].

## Authors' contributions

TM and BN designed the research; CB and JMB performed experiments and analyzed data; CB and BN wrote the paper. All authors read and approved the final manuscript.
